# Impact of heat shock step on bacterial transformation efficiency

**Published:** 2016-12

**Authors:** Maral Rahimzadeh, Majid Sadeghizadeh, Farhood Najafi, Seyed Arab, Hamid Mobasheri

**Affiliations:** 1 Department of Nanobiotechnology, School of Biological Sciences, Tarbiat Modares University, Tehran, Iran; 2 Department of Molecular Genetics, School of Biological Sciences, Tarbiat Modares University, Tehran, Iran; 3 Department of Resin and Additives, Institute for Color Science and Technology, Tehran, Iran; 4 Department of Biophysics, School of Biological Sciences, Tarbiat Modares University, Tehran, Iran; 5 5) Laboratory of Membrane Biophysics, Institute of Biochemistry and Biophysics, University of Tehran, Tehran, Iran

**Keywords:** *E. coli*, Artificial transformation, Heat shock, Transformation efficiency

## Abstract

CaCl_2_ treatment followed by heat shock is the most common method for artificial transformation. Here, the cells were transformed using CaCl_2 _treatment either with heat shock (standard protocol) or without heat shock (lab protocol) to comprehend the difference in transformation efficiency. The BL21 strain of *Escherichia coli* (*E. coli*) was being susceptible using CaCl_2 _treatment. Some Cells were kept at -80 ^o^C while the others were kept at 4 ˚C. Afterwards the susceptible cells were transformed using either standard or lab protocol. The transformation efficiency between cells experienced heat shock and those were not influenced by heat shock was almost the same. Moreover, regardless of transformation protocol, the cells kept at 4 ˚C were transformed more efficiently in compared to those were kept at -80 ^o^C.

## INTRODUCTION

Bacteria can naturally obtain new generic information through 3 various mechanisms; conjugation, transduction and transformation [1]. Transferring of DNA directly from one organism to another one is called conjugation but in transduction DNA transferring is occurred with the aid of bacteriophage. During transformation naked DNA is bounden to the cell surface and passed through wall- membrane complex [2]. Transformation can happen either naturally or artificially in bacteria. Natural transformation is a rare mechanism used by some bacterial cells to take up DNA from the environment. In artificial transformation, bacterial cells should be susceptible under certain laboratory conditions prior to transformation. There are two main methods for artificial transformation in bacteria; CaCl_2_ treatment followed by brief heat shock and electroporation [3, 4]. Either of methods has been modified during the past century to achieve more transformation efficiency.

The exact mechanism of how CaCl_2_ treatment could facilitate transferring DNA from extracellular environment into cytoplasm is still unrevealed. It is assumed that DNA molecules can be absorbed on the cell surface with the help of divalent cation Ca^2+ ^and heat shock step make entering DNA into cytosol possible [2].

In this study, *E. coli* bacteria were transformed using two methods; (1) CaCl_2_ treatment followed by heat shock step and (2) CaCl_2_ treatment without using heat shock step. The transformation efficiency was calculated for both methods. It seems that heat shock step may not have the crucial role for transformation protocol.

## MATERIALS AND METHODS

The BL21 strain of *E. coli* bacteria was used for transformation. The pEGFP-N1 vector originally purchased from Clontech Laboratories, Inc. (USA). The vector is kanamycin resistant and contains enhanced green fluorescent protein (EGFP) gene.

*E. coli* bacteria were grown in Luria Bertani (LB) medium. 15 µL of overnight cultured *E. coli* was inoculated to 25 ml LB broth and was incubated on a shaker at 37 ˚C and 250 rpm until the optical density of suspension at 600 nm reached in the range of 0.4-0.7. Then the suspensions were kept on ice for 30 min [5]. Following this step, the bacteria were pelleted using Dragon Lab centrifuge (D3024R, Dragon Laboratory Instruments Limited, China) at 1717 ×g for 20 min at 4 ˚C. The bacterial pellet was re-suspended in 12.5 ml CaCl_2 _(100mM) and placed on ice for 30 min. Next, the cell suspensions were centrifuged as above and pellets were dissolved in 25 ml CaCl_2_ (100mM). At this stage, suspensions kept on ice overnight. Again the bacteria were pelleted at 1717 ×g for 20 min at 4 ˚C and the pellets were re-suspended in 2250 µl CaCl_2_ (100mM) and 750 µl glycerol 60% and stored at -80 ˚C and/or 4 ˚C for further experiments.

Transformations were performed using either standard protocol or lab protocol as follows; 100 µl of thawed competent bacteria and 0.65 ng of pEGFP-N1 DNA (isolated from *E. coli *strain DH5-alpha with AccuPrep® plasmid mini extraction kit from Bioneer and qualified with gel agarose electrophoresis) were added to pre-chilled tube and pipetted gently. In standard protocol, the suspensions were kept on ice for 30 min and after that the mixture were heated at 42 ˚C for 45 seconds. Following heat shock step the tubes were kept on ice for another 5 min. Then 1 ml LB broth was added and serially diluted suspensions were prepared and incubated in a shaker at 37 ˚C for 2 hours. But in our lab protocol, the ice and heat shock steps were completely omitted and 1 ml LB broth was immediately added to the mixture of DNA and bacterial cells (at 25 ˚C) and the suspension was serially diluted and incubated in a shaker at 250 rpm for 2 hours at 37 ˚C. At the end of both protocols, the cells were centrifuged at 1717 ×g for 5 min. and pellets were re-suspended in 100 µl LB and streaked on kanamycin resistant plate.

The experiments were performed in quadruple. The data are reported as mean ± standard deviation. The unpaired t- test was performed in order to understand the significance of difference. The two-tailed P values were assigned less than 0.05. The GraphPad Prism 5 software was used for analysis.

## RESULTS AND DISCUSSION

The transformation was performed using cells kept at either 4 ˚C (fresh) or -80 ˚C with both standard and lab protocol. The cells were successfully transformed with standard and lab protocol. The number of bacteria which were able to uptake DNA molecules is defined as Transformation Efficiency (TE) [2]. The TE was calculated from Equation 1.

TE=number of colony forming units (CFU)the amount (in µg) of plasmid DNA

**Figure 1:**
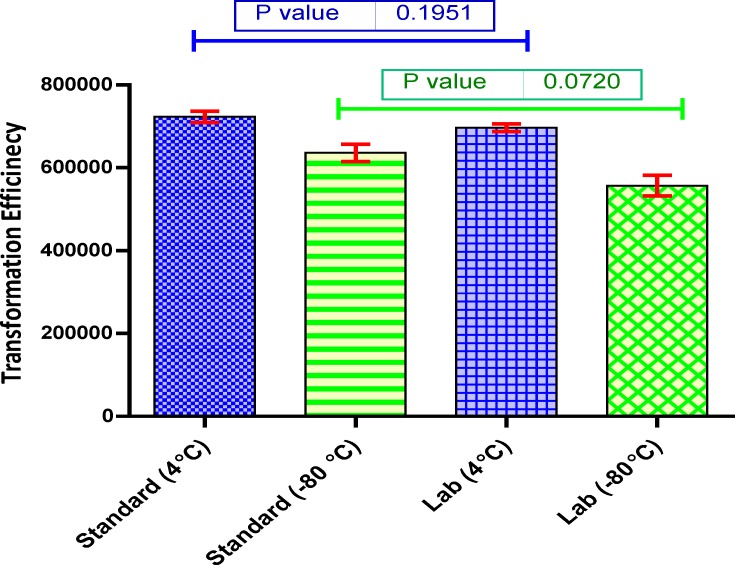
Transformation efficiencies of various conditions

**Figure 2:**
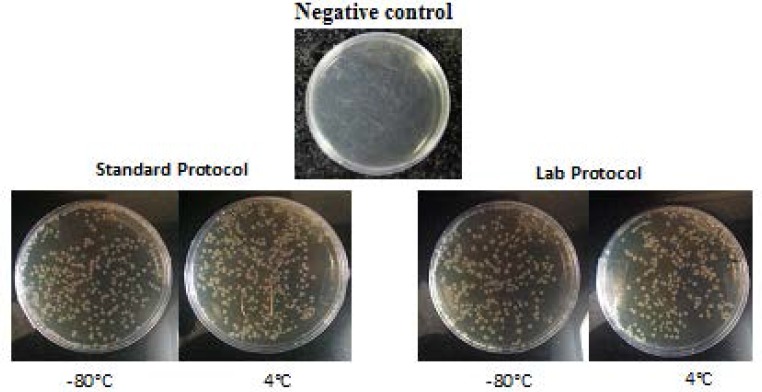
Successful diluted bacterial transformation plates using standard and lab protocol (The samples were diluted 100 times

In general, the TE of fresh cells (kept at 4 ˚C) was more than those were kept for longer period at -80 ^o^C. Notably, the difference between lab and standard protocol was quite low and bacteria could be transformed without applying heat shock. The transformation efficiencies are displayed in Figure 1. The successful transformation plates are presented in Figure 2.

It was suggested that heat shock step could facilitate DNA entry but still there is not enough clues. Panja et al (2006) reported that heat-pulse step cause reduction in membrane potential [2]. The cellular inside potential is became less negative as a result of membrane potential decrease therefore, the negative DNA could enter the cytosol easier [2].

Although the exact role of Ca^2+^ ions is not known yet, it is believed that Ca^2+^ could develop the interaction between DNA molecules and LPS (lipopolysaccharide) of outer membrane. Also, Ca^2+^ ions could alter the physio-chemical properties of lipids and induce phase transition of phosphatidylglycerol and LPS [6-8]. Furthermore, Ca^2+ ^divalent cations enhance structural changes in phosphatidylcholine-cardiolipin bilayers which lead to increased permeability [6, 9-11]. 

Our results suggest that Ca^2+^ ions play more crucial role in artificial transformation. Also, it seems that the ability of Ca^2+^ in increasing the membrane permeability is more than heat shock. At the same time, it should be noted that in the lab protocol cells were undergone a brief heat shock (0 →25 → 37 ˚C) which is appeared to be enough for transferring DNA. In order to understand the exact mechanism of Ca^2+ ^ions and heat shock step in transformation, more investigation need to be performed.
